# A Compact UWB Antenna with Independently Controllable Notch Bands

**DOI:** 10.3390/s19061411

**Published:** 2019-03-22

**Authors:** Amjad Iqbal, Amor Smida, Nazih Khaddaj Mallat, Mohammad Tariqul Islam, Sunghwan Kim

**Affiliations:** 1 Centre for Wireless Technology (CWT), Faculty of Engineering, Multimedia University, Cyberjaya 63100, Malaysia; aiqbal@ieee.org; 2 Department of Medical Equipment Technology, College of Applied Medical Sciences, Majmaah University, AlMajmaah 11952, Saudi Arabia; 3 Unit of Research in High Frequency Electronic Circuits and Systems, Faculty of Mathematical, Physical and Natural Sciences of Tunis, Tunis El Manar University, Tunis 2092, Tunisia; 4 College of Engineering, Al Ain University of Science and Technology, Al Ain 64141, UAE; nazih.mallat@aau.ac.ae; 5 Centre of Advanced Electronic and Communication Engineering, Faculty of Engineering and Built Environment, Universiti Kebangsaan Malaysia, Bangi 43600, Malaysia; tariqul@ukm.edu.my; 6 School of Electrical Engineering, University of Ulsan, Ulsan 44610, Korea

**Keywords:** meandered slots, resonators, patch antenna, S-parameters

## Abstract

A minimally-sized, triple-notched band ultra-wideband (UWB) antenna, useful for many applications, is designed, analyzed, and experimentally validated in this paper. A modified maple leaf-shaped main radiating element with partial ground is used in the proposed design. An E-shaped resonator, meandered slot, and U-shaped slot are implemented in the proposed design to block the co-existing bands. The E-shaped resonator stops frequencies ranging from 1.8–2.3 GHz (Advanced Wireless System (AWS1–AWS2) band), while the meandered slot blocks frequencies from 3.2–3.8 GHz (WiMAX band). The co-existing band ranging from 5.6–6.1 GHz (IEEE 802.11/HIPERLANband) is blocked by utilizing the U-shaped section in the feeding network. The notched bands can be independently controlled over a wide range of frequencies using specific parameters. The proposed antenna is suitable for many applications because of its flat gain, good radiation characteristics at both principal planes, uniform group delay, and non-varying transfer function ($S_{21}$) for the entire UWB frequency range.

## 1. Introduction

Currently, there is significant interest in ultra-wideband (UWB) technology, as it is considered to be an energy-efficient choice for short-range communication. One of the important radio frequency (RF) front-end components of such a communication system is the antenna [1,2,3,4]. The antenna should be designed with qualities such as compactness and good performance [5]. UWB technology is preferred in many emerging technologies involving multiple-input multiple-output (MIMO) [6,7], ground-penetrating radar [8,9], health care [10], and the Internet of Things [11].

A typical method for avoiding interference between the newly-constructed system and co-existing communication standards is to associate a stop band filter with the UWB system to block the interfering frequency range. However, this method consumes excessive room and increases the outline intricacy significantly [5]. Another convenient method for removing existing interference from the current system is to implement frequency blocking designs in the proposed structure in order to stop the undesired frequencies. Two important challenges while designing these structures are the shape and placement [12,13,14]. Previous studies have introduced a large number of UWB printed antennas with band-notched functionality composed of a single stop-band [15], dual stop-band [16], or multiple stop-bands [17] for the proposed frequency bands. To produce notches at the undesired frequency bands, a split ring-shaped slot and split ring resonator were introduced in the UWB antenna [18]. An open loop resonator was used as a frequency notch structure in [19] to stop a single frequency band in UWB antenna. Multiple notch bands were obtained in [20] by introduction of C-shaped stubs. Furthermore, crescent-shaped resonators on the ground plane can generate multiple notches by being shorted with the radiating element through plated holes [21]. A single notch in a co-planar waveguide (CPW)-fed UWB antenna was created using defected ground plane [22]. Multiple notches in a CPW-fed UWB antenna were created using a meandered line resonator [23]. A number of antennas have been presented recently incorporating controlled rejected bands over a wide range of frequencies. In [24], a dual-notched band antenna was presented with controllable rejected bands. In [25], a controllable single rejected band was achieved in a UWB antenna with different notched frequencies and notch amplitude choices. The antennas discussed in the literature have good notch characteristics and compact sizes, but they do not allow for independent adjustment of multiple notches.

In this work, an independently-controllable notched band UWB antenna is presented for many useful applications. In the reported antenna, a notch band at AWS1–AWS2 (1.8–2.3 GHz) is obtained by placing a modified E-shaped stub on the opposite side of the patch; a second notch at the WiMAX band (3.2–3.8 GHz) is achieved by creating a meandered slot in the patch; and the third notch at the IEEE 802.11/HIPERLAN band (5.6–6.1 GHz) is obtained by creating a U-shaped slot in the transmission line of the antenna. The proposed antenna has four passbands: 1.4–1.79 GHz, 2.31–3.19 GHz, 3.81–5.59 GHz, and 6.12–11.3 GHz. The notching structures are placed so as to have very little impact on each other, allowing for independently-controllable rejected bands. An equivalent circuit model of the UWB antenna is designed and matched with the EM model.

## 2. Design Methodology

**Figure 1:**
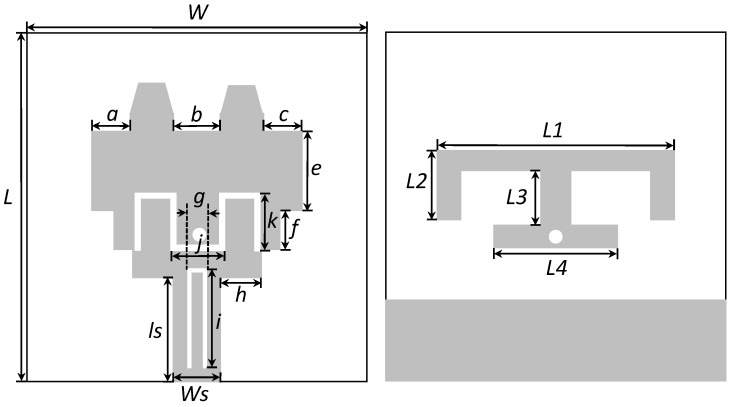
Labeled schematic of the proposed antenna.

The proposed UWB antenna is shown in Figure 1 and its associated parameters are listed in Table 1. A step-by-step analysis of the proposed antenna is performed so as to assess the performance of the antenna at each step. The aim of including this section is to clarify the method for achieving the three notched bands at co-existing frequencies and the effect of each notch-creating structure on the performance of the reference antenna. Each step indicates the antenna behavior in terms of voltage standing wave ratio (VSWR) and input impedance. The equivalent circuit model in each step is designed and matched with the input impedance of the EM model. The values of the optimized equivalent circuit model are listed for each step.

### 2.1. Step 1: Ultra-Wideband Antenna

**Figure 2:**
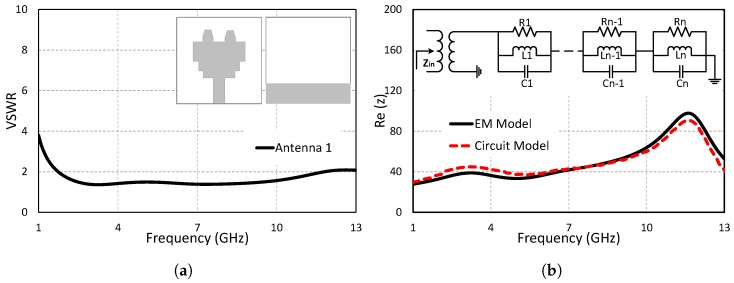
Step 1: Ultra-wideband (UWB) antenna, (**a**) voltage standing wave ratio (VSWR). (**b**) Input impedance (z).

A printed antenna consisting of the modified maple leaf-shaped main radiator and partial ground is used as the UWB antenna. FR-4 ($\epsilon_{r}$ = 4.4, *h* = 1.6 mm) material is used as a substrate for the proposed design. The overall dimensions of the antenna are 33 × 34 × 1.6 mm${}^{3}$. A 50 Ω transmission line is designed with a width of 3 mm for the purpose of transferring maximum power to the proposed printed UWB antenna. The bandwidth of the rectangular patch is enhanced by cutting its lower edge to get the UWB performance. The structure of the designed UWB antenna is shown in Figure 2a. The VSWR of the UWB antenna is shown in Figure 2a against varying frequencies. The VSWR values range between 1 and 2, showing perfect impedance matching for the entire UWB frequency range. The designed UWB antenna operates over the entire UWB range (1.4 GHz–11.3 GHz).

One of the significant difficulties in improving UWB networks is the co-structure of UWB antennas with other RF front-end elements. To understand the true performance of the system, it is always necessary to perform co-simulation of the UWB antenna with the other RF front-end elements. It is necessary to determine the equivalent circuit model of the antenna because most of the RF front-end elements are analyzed in time domain simulators, such as the Advance Design System (ADS) and SPICE. The key factor of the equivalent circuit model is that its input impedance should be well matched with the modeled antenna. Figure 2b shows the equivalent circuit diagram [26] and input impedance of the antenna. The wideband frequency range of the UWB antenna is due to the large number of independent resonances overlapping with one another. Using the same concept, the proposed UWB antenna is modeled with multiple RLC components connected in series. The results show that the input impedance of the equivalent circuit model is well matched with the EM model of the antenna over the entire bandwidth.

### 2.2. Step 2: Single Band-Notched UWB Antenna

**Figure 3:**
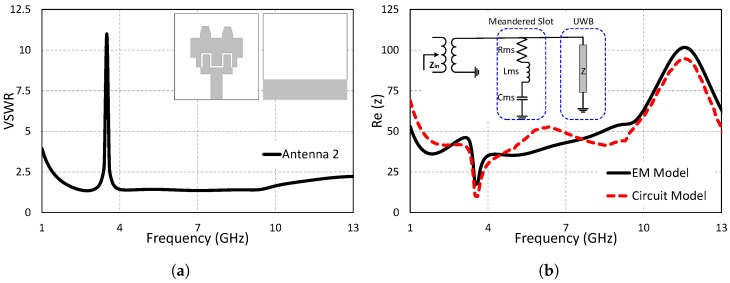
Step 2: Single band-notched UWB antenna. (**a**) VSWR. (**b**) Input impedance (z).

One of the critical issues of a band-notched antenna is the placement of its notch-creating structures in the antenna elements to stop the co-existing bands. The meandered slot resonator is placed in the main radiator in order to block the co-existing band centered at 3.5 GHz (WiMAX), covering the frequency range from 3.2 GHz–3.8 GHz. The single-notched band antenna is shown in Figure 3a. It is important to note that the dimensions of the previously designed UWB remain unchanged when the notching structure is added to the antenna. Hence, no retuning is required for the existing UWB antenna designed in Step 1. The dimensions of the meandered slot are calculated using the following design equations.

(1) $$f_{r}=\frac{1}{2L\sqrt{\epsilon_{eff}}}$$

(2) $$\epsilon_{eff}=\frac{\epsilon_{r}+1}{2}$$

where $f_{r}$ is the resonant frequency, $\epsilon_{r}$ is the relative permittivity, and *L* is the slot’s length. The theoretically-calculated meandered slot value for 3.5 GHz is 43 mm, while the optimized value for the meandered slot is noted as 41.4 mm. The position of the meandered slot is adjusted by parametric analysis for the best results. Figure 3b shows the equivalent circuit model of the single-notched band UWB antenna. Notching structures are designed by utilizing a conceptual circuit model and connecting it with the antenna input impedance as a series or parallel RLC circuit, keeping in view the impedance at co-existing bands. A series RLC circuit is connected in parallel with the input impedance for the notched band centered at 3.5 GHz. The values of the equivalent circuit model (*Rms* = 25 Ω, *Lms* = 0.5 nH, *Cms* = 30.85 pF) are determined by using ADS. The input impedance of the equivalent circuit model, as well as the EM model is shown in Figure 3b.

### 2.3. Step 3: Dual Band-Notched UWB Antenna

**Figure 4:**
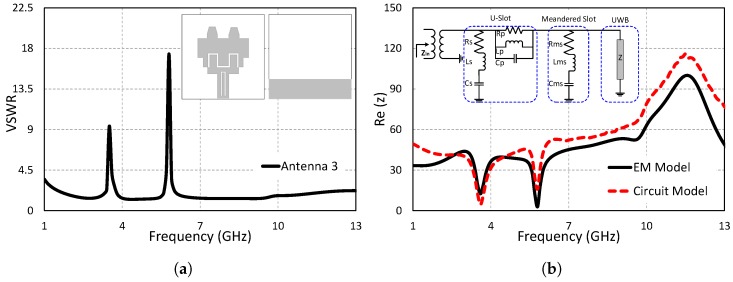
Step 3: Dual band-notched UWB antenna. (**a**) VSWR. (**b**) Input impedance (z).

To overcome the unwanted potential interference of the narrow band system with the UWB antenna, a second stop band in addition to the WiMAX band is generated at 5.8 GHz (IEEE 802.11/HIPERLAN), covering the frequency range from 5.6 GHz–6.1 GHz. A U-shaped slot is etched at the microstrip transmission line to stop the IEEE 802.11/HIPERLAN band. The length of the U-shaped slot is calculated as 20.4 mm using (1), while the optimized value is obtained as 20.5 mm. The structure of the dual band-notched antenna and its VSWR results are shown in Figure 4a. The VSWR result indicate that a second band is introduced in the existing single band-notched antenna without deteriorating the performance of the single band-notched antenna. The equivalent circuit model is successfully designed by combining the series RLC resonant circuit and parallel RLC resonant circuit into the single band-notched antenna presented in Figure 4b. The impedance values of the equivalent circuit model and EM model matched reasonably. The two dips in the impedance graph of both models (EM and equivalent circuit model) represent the notched bands. The optimized values of the equivalent circuit model (*Rs* = 25 Ω, *Cs* = 0.5 pF, *Ls* = 100.5 nH, *Rp* = 175.325 Ω, *Cp* = 122.2 pF, *Lp* = 160.1 nH) are obtained through ADS.

### 2.4. Step 4: Triple Band-Notched UWB Antenna (Proposed Antenna)

**Figure 5:**
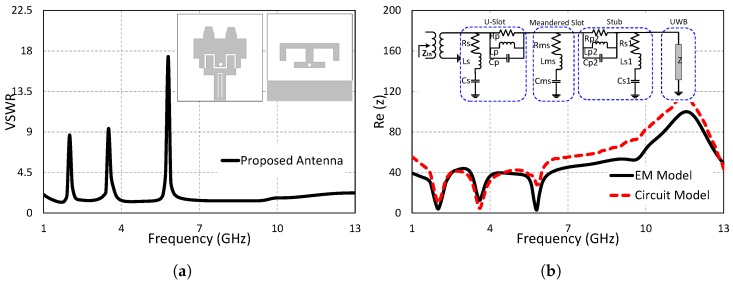
Step 4: Triple band-notched UWB antenna (proposed antenna). (**a**) VSWR. (**b**) Input impedance (z).

A modified E-shaped stub resonator is designed on the bottom of the substrate to mitigate the interference of the UWB antenna with the co-existing narrow band system at the AWS1–AWS2 band by blocking the same band of the UWB antenna. The E-shaped stub resonator is connected to the radiating patch through a 2 mm circular via post. The introduction of the modified E-shaped resonator results in the blockage of the AWS1–AWS2 band (1.8–2.3 GHz) without affecting the performance of the dual band-notched UWB antenna. The structure and VSWR results of the triple band-notched (proposed) antenna are shown in Figure 5a. The proposed antenna has a wide bandwidth, as well as good notching characteristics at the co-existing bands. The labeled diagram of the proposed antenna is presented in Figure 1. The detailed dimensions of the antenna’s associated parameters are shown in Table 1. The equivalent circuit model is designed as a combination of series and parallel RLC resonant circuits. Good agreement between the impedance results of the EM model and equivalent circuit model is observed (Figure 5b). The three dips at 2 GHz, 3.5 GHz, and 5.8 GHz in the impedance graphs show the mismatch at these frequencies, resulting in creating notches at the corresponding frequencies.

## 3. Parametric Analysis

A detailed parametric analysis of the proposed antenna was carried out so as to assess the performance of the antenna and to determine the control parameters of each notch band. The control of the notch bands is required in practical applications of UWB antennas. However, the modern wireless communication architecture demands for independent controllable notched UWB antennas. A detailed study of the parameters of the notching structure is required to assess the best parameters for controlling the notches. The influences of the notching parameters on each notch were studied thoroughly, while the rest of the parameters are kept unchanged.

### 3.1. Controlling the Rejected AWS1–AWS2 Band (1.8–2.3 GHz)

**Figure 6:**
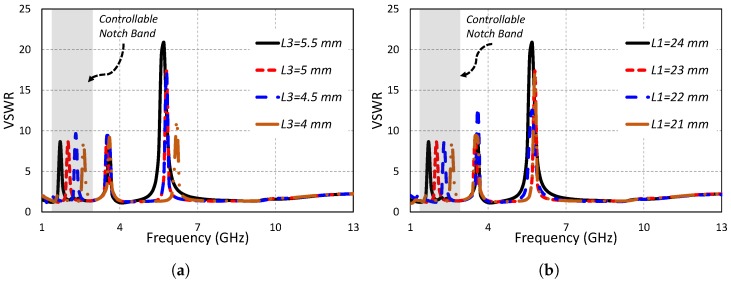
Independently-controllable lower notch band by varying (**a**) *L3* and (**b**) *L1*.

Figure 6a,b show the antenna behavior in terms of VSWR for a range of values of the parameters *L3* and *L1*, respectively. *L3* and *L1* are the notch-controlling parameters for the lower band (AWS1–AWS2) of the proposed antenna. The lower frequency notch band was centered at 2 GHz; the middle frequency notch was centered at 3.5 GHz; and the higher frequency notch was centered at 5.8 GHz for the optimized antenna (*L3* = 5 mm and *L1* = 23 mm). When the value of parameter *L3* was changed from 5 mm to 5.5 mm, the lower notch band shifted towards the lower frequency side, while the middle notch band and higher notch band remained unchanged; however, a slight increase in the bandwidth of the higher notch band was observed. The lower frequency notch was observed at 1.7 GHz for *L3* = 5.5 mm. Hence, a 300 MHz shift towards the lower frequency side was observed as a result of changing the length of *L3* = 5 mm by 0.5 mm. When the length of *L3* was decreased from 5 mm to 4.5 mm, the lower notch band shifted from 2 GHz to 2.3 GHz. The remaining two notch bands remained unaffected by this decrement. By further decreasing the value of the parameter *L3* from 4.5 mm to 4 mm, the lower frequency notch shifted from 2.3 GHz to 2.6 GHz. Furthermore, a shift to higher frequency in the higher frequency notch was observed for this change, while the middle notch band was resistive to parameter *L3* and remained unaffected under all scenarios. In the same manner, changing the parameter *L1* affected only the lower notched band, while the other notched bands remain unchanged. Changing the value *L1* from 23 mm to 24 mm shifted the lower notch band from 2 GHz to 1.7 GHz, while the other two notch bands remained unchanged. By reducing the value of parameter *L1* from 23 mm to 22 mm, the lower notch band shifted 300 MHz towards the higher frequency side, while the middle and the higher frequency notch band remained unaffected. Further reducing the value of *L1* from 22 mm to 21 mm caused the lower notch band to shift from 2.3 GHz to 2.55 GHz, inducing no effect on the middle and higher notch bands. It can be concluded from the parametric analysis of the parameters *L3* and *L1* that the lower notch band can be independently controlled by adjusting the parameters *L3* and *L1*.

### 3.2. Controlling the Rejected WiMAX Band (3.2–3.8 GHz)

**Figure 7:**
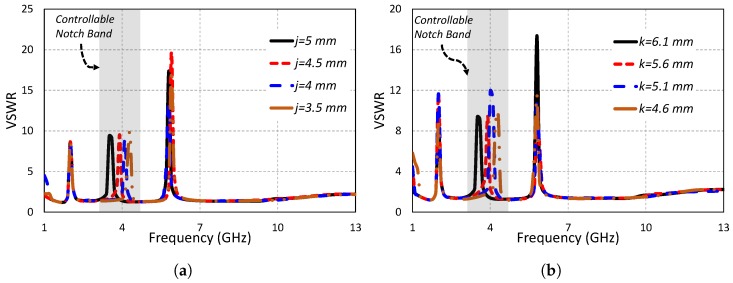
Independently-controllable middle notch band by varying (**a**) *j* and (**b**) *k*.

The meandered slot in the radiating part of the antenna is responsible for the notch generation at the WiMAX band. Figure 7a,b show the VSWR plot for variations in the parameters *j* and *k*, respectively. It is clear from both graphs that these two parameters are responsible for controlling the notch at the WiMAX band. For *j* = 5 mm, the notch at WiMAX band lied at 3.5 GHz. By decreasing the value of *j* from 5 mm to 4.5 mm, the middle notch (WiMAX) shifted from 3.5 GHz to 3.9 GHz. By further decreasing the value of *j* to 4 mm, the middle notch shifted to 4.2 GHz. The notch frequency shifted to a higher frequency (4.3 GHz) when the parameter *j* was decreased to 3.5 mm. In all cases, the lower and higher notches remained unaffected by changes in the parameter *j*. In the same way, changing the value of parameter *k* affected only the middle notch band, while the other two notch bands remained unchanged. At *k* = 6.1 mm, the middle notch band existed at 3.5 GHz. By changing the parameter *k* value to 5.6 mm, the middle notch shifted to 3.9 GHz. Further decreasing the value of *k* to 5.1 mm shifted the middle notch to 4.11 GHz. At *k* = 4.6 mm, the center of the middle notch lied at 4.3 GHz. It is concluded from studying the parametric analysis of *j* and *k* that the middle notch can be independently controlled by adjusting these two important parameters. It is also observed that these two parameters have no influence on the lower frequency (AWS1–AWS2) and higher frequency (IEEE 802.11/HIPERLAN) notch bands.

### 3.3. Controlling the Rejected IEEE 802.11/HIPERLAN Band (5.6–6.1 GHz)

**Figure 8:**
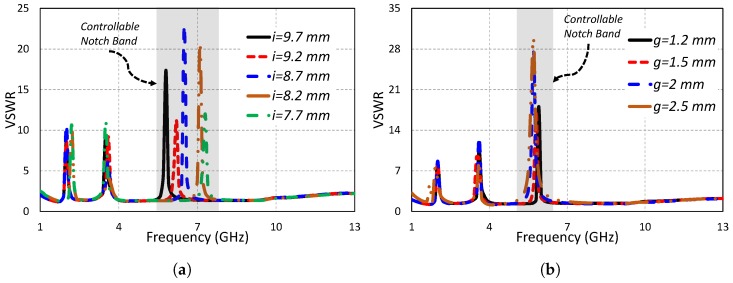
Independently-controllable higher notch band by varying (**a**) *i* and (**b**) *g*.

Just like the controllable parameters of AWS1–AWS2 notch band and WiMAX notch band, parameters *i* and *g* are the controllable parameters of the IEEE 802.11/HIPERLAN notch band. Figure 8a,b show the antenna VSWR for changing parameters *i* and *g*, respectively. Changing the value of the parameter *i* changed the higher frequency notch band, while the lower and middle frequency notch bands remained unchanged. It is shown in Figure 8a that decreasing the value of parameter *i* shifted the higher notch band towards higher frequencies. The higher frequency notch lied at 5.8 GHz for *i* = 9.7 mm and shifted to 6.2 GHz for *i* = 9.2 mm. The higher band notch shifted to 6.5 GHz for 8.7 mm. By further decreasing the U-shaped slot length (*i*) to 8.2 mm, the notch band shifted to 7.1 GHz. For *i* = 7.7 mm, the center of the higher notch band was at 7.3 GHz. It is concluded from the parametric study of the parameter *i* that *i* has a huge impact on the positioning of the higher frequency notch band, while the rest of the notch bands remain unchanged. In the same way, the parameter *g* also affected only the higher frequency notch band. Changing the value of the parameter *g* changed only the higher frequency notch band. Increasing the value of *g* shifted the higher notch band towards lower frequencies. It is concluded from studying the parametric analysis of *i* and *g* that the higher frequency notch band can be independently controlled by adjusting these two important parameters.

## 4. Results and Discussion

**Figure 9:**
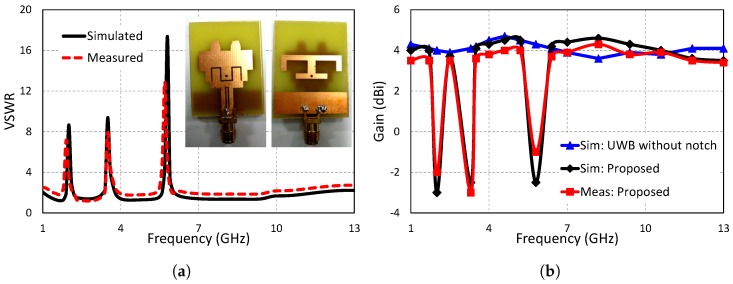
Simulated and measured (**a**) VSWR and (**b**) peak gain.

To demonstrate the validity and performance of the design, an antenna prototype was produced and subjected to experimental measurements. Figure 9a shows the fabricated antenna based on the aforementioned parameters.

### 4.1. VSWR Measurement

Vector Network Analyzer (VNA) model HP 8720D (50 MHz–13.5 GHz) was used for the validation of the proposed antenna’s VSWR. The proposed antenna model (Figure 9a) was tested, and its measured results were obtained by connecting it with the VNA. The simulated results were in good accordance with the measured results, as shown in Figure 9a. The results indicate that the designed model effectively rejects three frequency bands (1.8–2.3 GHz (AWS1–AWS2), 3.2–3.8 GHz (WiMAX), and 5.6–6.1 GHz (IEEE 802.11/HIPERLAN)), while preserving wideband performance from 1.4–11.3 GHz with VSWR values between one and two.

### 4.2. Peak Gain

The results of gain obtained from simulating the model in High Frequency Structure Simulator (HFSS 13.0) software were found to be in good agreement with measured results obtained from the fabricated prototype of the antenna, as shown in Figure 9b. As appears in Figure 9b, the proposed UWB notched antenna approximately followed the gain of the reference antenna and had almost flat gain over the entire operating range. However, three sharp reductions in the gain, representing the three notch bands, can be observed (Figure 9b). For the reference antenna, the maximum peak gain was 4.7 dBi at 4.6 GHz, and the minimum gain was observed as 3.6 dBi at 8.2 GHz. The simulated maximum gain of the proposed antenna was observed as 4.6 dBi, while the maximum gain of the measured results was found to be 4.35 dBi.

### 4.3. Surface Current Distribution (Jsurf)

**Figure 10:**
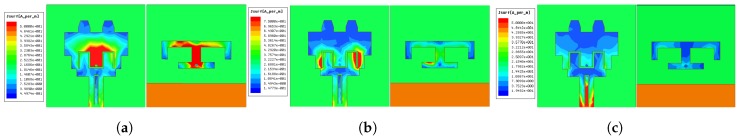
Surface current distribution at (**a**) 2 GHz, (**b**) 3.5 GHz, and (**c**) 5.8 GHz.

The effect of the notching structure can be evaluated through the analysis of the antenna’s surface current distribution, as shown in Figure 10. At the 2 GHz notch band, most of the current is concentrated on the back side of the modified E-shaped resonator, and a nearly uniform current is distributed on the remaining parts of the antenna. The antenna becomes unresponsive for the given band because of the destructive interference of current caused at the modified E-shaped resonator. A strong current distribution is seen at the meandered slot for the 3.5 GHz notched band, which further clarifies that this notch is generated because of the meandered slot. In the same way, destructive interference is caused by the meandered slot at 3.5 GHz, and it is rejected. The surface current distribution at 5.8 GHz is shown in Figure 10c. It is clear from Figure 10c that high current density was concentrated at the U-shaped slot on the transmission line, which shows that the U-shaped slot was responsible for generating the notch at 5.8 GHz.

### 4.4. Radiation Pattern

**Figure 11:**
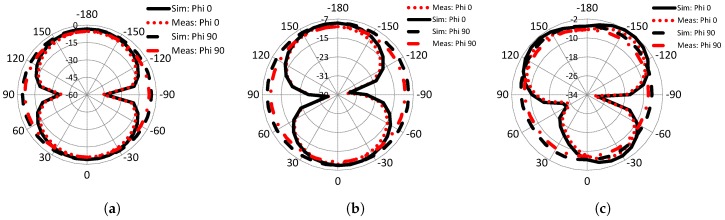
Simulated and measured radiation pattern at (**a**) 1.6 GHz, (**b**) 2.66 GHz, and (**c**) 7.2 GHz.

Figure 11a–c portray the simulated and measured far-field radiation patterns in both principal planes $\phi =0^{{}^{\circ}}$ and $90^{{}^{\circ}}$ for 1.6 GHz, 2.66 GHz, and 7.2 GHz, respectively. The results obtained from simulating the model in HFSS software were found to be in good agreement with the measured results obtained from the fabricated prototype of the antenna. As depicted in these figures, the radiation patterns were omnidirectional in the E-plane and a monopole-like pattern in the H-plane at the frequencies of interest, which makes it a competitive choice for many sensing applications. For higher frequencies, the radiation patterns in both planes were distorted because of the non-uniform phase division and higher order modes. The radiation pattern in the E-plane can be improved using a low permittivity or slim substrate and by keeping the ground plane equal to the radiating element [27].

### 4.5. Group Delay and Transfer Function

**Figure 12:**
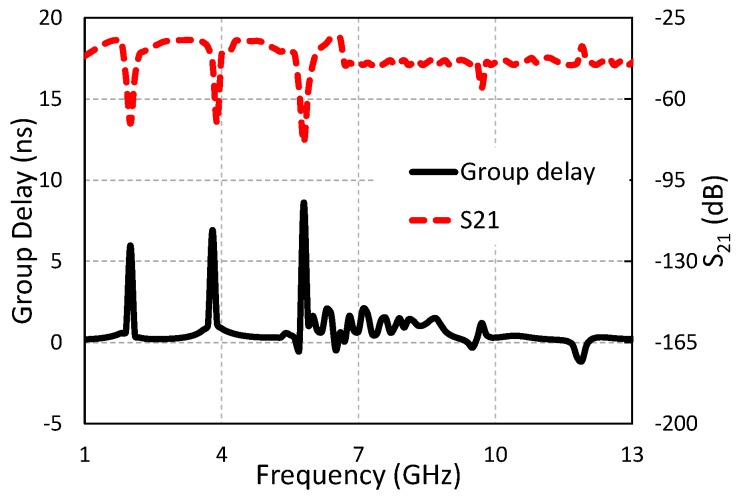
Group delay and transfer function of the triple band-notched UWB antenna.

The degree of distortion in the UWB antenna can be characterized using group delay. A stable group delay with less non-uniformity is always desirable for the whole UWB frequency range in a UWB system. To create a far-field situation, two similar antennas are placed face to face at a distance of 30 cm. Figure 12 shows the group delay of the proposed UWB antenna. The group delay of the proposed antenna is somehow uniform, except at the notch bands. The group delay has less variation (<2 ns) over the UWB frequency range except at the notch bands (>6 ns). It can be concluded from Figure 12 that the signal of the UWB system was not distorted between the transmitting and the receiving antenna. The transfer function ($S_{21}$) also showed less distortion, except at the three rejected bands (Figure 12). The flat group delay and transfer function make this antenna suitable for many useful applications.

The proposed UWB antenna is compared with state-of-the-art UWB antennas in Table 2.

## 5. Conclusions

The design, prototyping, and measurements related to a modified maple leaf-shaped UWB antenna with three notch bands have been thoroughly depicted. The proposed antenna successfully rejects three co-existing bands while keeping good performance over the entire passband. Notches in the proposed design were generated by optimizing the notching structures in the radiation element and feeding line and placing a modified E-shaped stub resonator on the bottom of the substrate. The three notch bands of the antenna can be independently tuned using their respective controllable parameters.

## Figures and Tables

**Table 1:** Different parameters and values of the antenna.

Parameter	Value (mm)	Parameter	Value (mm)	Parameter	Value (mm)
*a*	4.5	*g*	1.5	*L3*	5
*b*	4	*h*	4.04	*L4*	12
*c*	4.5	*i*	8	*L*	34
*e*	7.85	*j*	5.1	*L2*	6
*f*	4.31	*k*	5.6	*ls*	10.3
*Ws*	3	*W*	33	*L1*	11.5

**Table 2:** Comparison with previously published work.

Ref.	Size (mm^3^)	Notch Bands (GHz)	Frequency Coverage (GHz)	VSWR${}_{\max}^{*}$	Max. Gain (dBi)	Notch Controlling
[2]	40.4 × 44 × 0.1	5.25, 5.775	3–11	9.8, 8	2–4	No
[3]	36 × 34 × 1	3.5, 5.2, 5.8	2.9–13	>10	4–5	Yes, but partially
[12]	24 × 35 × 1	3.6, 5.7, 7.3	3.1–11.0	8.5, 8, 5.5	02–08	No
[15]	30 × 35 × 1.6	5.8	3.1–11.15	NG${}^{*}$	3.02	No
[18]	34.5 × 38.31 × 1.6	3.54, 5.24, 5.85	3.1–10.6	36, 22, 6	02–05	No
[24]	30 × 28 × 0.8	5–5.8	3–11	25	2.5–7	Yes
[25]	15 × 15 × 0.5	5.8	2.8–12	20	NG${}^{*}$	Yes
**This work**	**33 × 34 × 1.6**	**2, 3.5, 5.8**	**1.4–11.3**	**9, 10, 17**	**3.6–4.6**	**Yes**

VSWR${}_{max}^{*}$ shows the maximums of the notch bands, NG${}^{*}$ = not given.
